# Acute Symptomatic Calcific Discitis Mimicking a Septic Spondylodiscitis

**DOI:** 10.1155/2020/5454197

**Published:** 2020-03-09

**Authors:** Magda Choueiri, Florent Eymard, Sandra Guignard, Frédéric Pigneur, Xavier Chevalier

**Affiliations:** ^1^Department of Rheumatology, Henri Mondor University Hospital, Paris XII University-UPEC, Créteil, France; ^2^Department of Radiology, Henri Mondor University Hospital, Paris XII University-UPEC, Créteil, France

## Abstract

Acute symptomatic calcific discitis is a poorly understood condition that has been mostly reported in children. Cases in adults have been scarcely reported and may mimic an infectious process. Imaging, including computed tomography, can show the disc calcification but might fail to show it because its resorption can occur early after the onset of symptoms. We report the case of an adult patient presenting with severe cervical-dorsal junction pain, fever, high C-reactive protein (CRP) levels, and imaging findings mimicking an infectious spondylodiscitis, including an erosion of the anterior part of the vertebral endplate. However, the patient improved spontaneously and rapidly, with pain and fever disappearing and C-reactive protein (CRP) returning to normal within a week.

## 1. Introduction

Acute symptomatic calcific discitis (CD) is a rare condition in the adult population, with very few cases reported in the literature [1]. Incident disc calcification is reported in 5% of chest radiographs and 6% of abdominal plain films, as well as in 70% of postmortem adult examinations [2]. However, these calcifications generally remain asymptomatic, but when they do manifest, they can induce acute onset severe spinal pain and stiffness, with imaging features mimicking infectious spondylodiscitis [1–8]. On the other hand, pyogenic spondylodiscitis can be extremely severe and is associated with high mortality rates ranging from 2 to 20% in the literature, which makes it a potentially life-threatening disease [9]. Hence, identifying those cases is crucial, and acknowledging plausible differential diagnosis can be quite helpful.

## 2. Case Report

A 67-year-old man presented to the emergency department for a two-week debilitating low cervical and high dorsal pain, with fever and asthenia. The pain was severe, throbbing, and occurring in small spontaneously remitting crisis, with an inflammatory pattern. There were no other symptoms, and no clinical signs of infection that could explain his fever. The patient was morbidly obese and nonsmoker and had a history of paroxysmal atrial fibrillation treated with digoxin, a beta-blocker, and warfarin and type II diabetes mellitus for which he was taking glimepiride; on presentation, his blood glucose level was moderately elevated (162 mg/dL, normal lab value (*N*) < 110 mg/dL) and his latest glycated hemoglobin levels were also slightly increased, reaching 6.8%.

At the emergency department, the patient was afebrile. Upon examination, he had a stiff cervical spine with pain on flexion. Palpation of his spine showed exquisite pain at the levels of C7 and T1. The remaining physical examination revealed no abnormalities including a normal neurologic state and a normal cardiac auscultation.

**Figure 1:**
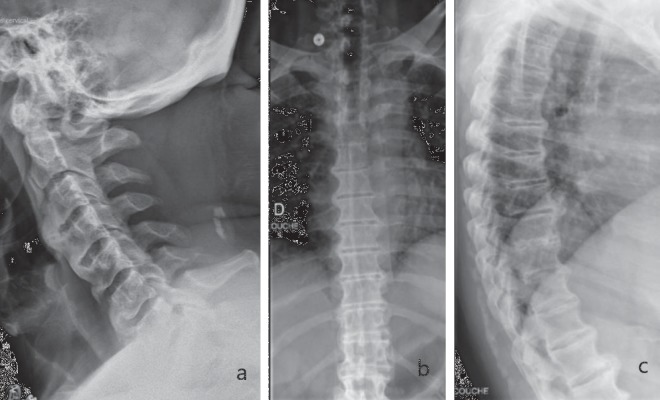
Plain radiographs of cervical spine, lateral view (a), and dorsal spine, anteroposterior (b) and lateral (c) views. Findings suggestive of diffuse idiopathic skeletal hyperostosis (DISH or Forestier's Disease) were noted, especially on dorsal spine X-rays. Calcification of intervertebral discs was not seen.

Laboratory evaluation showed a very high CRP level (306 mg/L, with *N* < 5 mg/L) and an increased erythrocyte sedimentation rate (68 mm after 1 hour, with *N* < 21 mm). His blood count and liver and renal function tests were normal. Chest X-ray revealed no abnormalities. Standard radiographs of the spine showed signs of diffuse idiopathic skeletal hyperostosis (DISH) with pseudoankylosis of the dorsal spine (Figure 1). No clear image of intervertebral calcific deposition was detected at this point.

**Figure 2:**
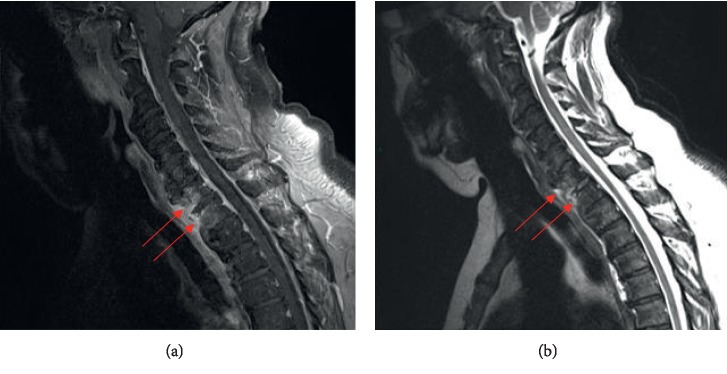
MRI of the cervico-dorsal junction, T1-weighted post-gadolinium injection (a) and T2-weighted (b) sequences, showing enhancement in C7 and T1 vertebral bodies after injection, and hyperintense signal on T2 sequence (arrows). The intervertebral C7-T1 disc does not show any T2 hypersignal or enhancement following contrast injection.

**Figure 3:**
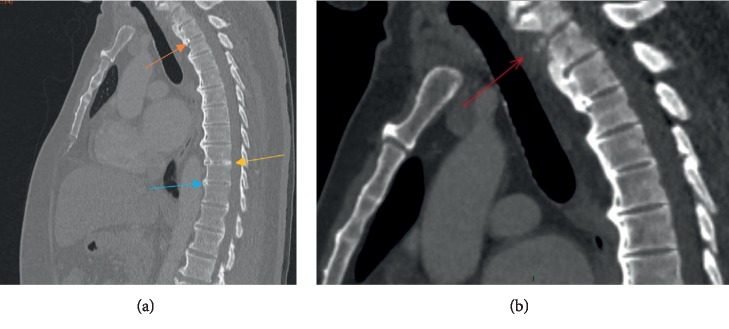
CT scan of the cervical and dorsal spine showing erosions on the anterior aspects of the lower vertebral endplate of C7 and the upper vertebral end-plate of T1 with a calcification located in between, at the anterior part of the C7-T1 intervertebral disc ((b) red arrow). (a) Calcifications were also present in the T2-T3 (orange arrow), T9-T10 (yellow arrow), and T10-T11 (blue arrow) intervertebral discs.

Owing to the strong suspicion of infectious spondylodiscitis, a magnetic resonance imaging (MRI) of the spine was performed (Figure 2) and showed inflammatory changes of both C7 and T1 vertebral bodies, with hyperintense signal on T2 and low signal intensity on T1 sequences, considerably enhancing after gadolinium injection. The intervertebral C7-T1 disc showed no T2 hypersignal and no enhancement following contrast injection. A computed tomography (CT) of the spine (Figure 3) showed erosions on the anterior aspects of the lower vertebral endplate of C7 and the upper vertebral endplate of T1 with a calcification located in between, at the anterior part of the C7-T1 intervertebral disc. Calcifications were noted in the adjacent disc spaces of the dorsal spines.

An echocardiography showed no sign of endocarditis. Urine and blood cultures, Wright serology, *Bartonella*, Q fever, hepatitis B and C, and HIV serologies were all negative. Three additional sets of blood cultures were taken during the first 24 hours of hospitalization and were also negative. Radiographs of the knees, the wrists, the shoulders, and the pelvis showed no signs of calcium pyrophosphate deposition.

A disco-vertebral biopsy was programmed, but it had to be delayed because the patient was taking warfarin for atrial fibrillation. In the meantime, since he remained stable, he received no antibiotics; no specific treatments, such as corticosteroids, were started. He was only prescribed analgesics and bed rest. Surprisingly, he started feeling better and his fever disappeared after a couple of days. His pain progressively decreased and had completely vanished a week later. His spine had retrieved a normal and painless mobility. Furthermore, his CRP levels dramatically dropped to 21 mg/L five days later and reached 14 mg/L seven days after his first consultation to the emergency department (vs. 306 mg/L on admission, *N* < 5 mg/L). No additional procedures were performed, and the patient was discharged.

This spectacular improvement in both clinical signs and biologic tests definitively ruled out the possibility of an infectious process. The diagnosis of acute symptomatic calcific discitis was made, accounting the acute clinical presentation and good evolution, along with the presence of calcifications in the several disc spaces of the dorsal spine.

## 3. Discussion

Although it has been frequently reported in children, acute symptomatic CD is a rare condition in the adult population, with no more than 10 cases reported in the literature [1].

The real etiology of CD remains unclear, with multiple possible theories. The most common hypothesis is the presence of a vascular insult, which could eventually lead to discal calcification by interrupting blood supply to the nucleus pulposus [10]. Repeated microtrauma might additionally play a role in this poorly understood process [1, 11]. Disc calcifications have also been observed in ankylosis of the spine.

Of note, conditions and metabolic diseases have been associated with intervertebral disc calcification, especially calcium pyrophosphate dihydrate deposition. These include hyperparathyroidism, hemochromatosis, ochronosis, amyloidosis, acromegaly, and poliomyelitis [12]. However, in these cases, calcification usually involves several levels, including at least the annulus fibrosus. This contrasts with idiopathic CD which only affects the nucleus pulposus and where no underlying systemic condition is usually found [1].

Interestingly, most case reports of CD occurring in adults show that the commonest locations of CD were the middle and lower thoracic spine, while the cervical localization is more frequently encountered in children. Indeed, among the few reported cases in adults, nine were located between T6 and T12 [1, 13], one was in C2-C3 [7], and one was in L4-L5 [14]. Our case is the first reported CD to occur in the cervico-thoracic junction in an adult patient. Another difference is the predominance of CD in boys contrasting with the adult population where it seems to occur more commonly in women [1]. Moreover, unlike adults, in whom no preceding event has been reported, children with CD often have a prior history of trauma (up to one third of cases) [14] or infection (15% of cases) [1, 15].

Fever and biologic signs of inflammation are usually absent in the few adult patients in whom CD has been reported. On the other hand, in the pediatric population, CD may present with fever and laboratory signs of inflammation (especially high CRP levels), which are detected in up to 15% of children with CD [2].

Hence, our case is the first report of acute symptomatic calcific discitis associated with fever and biological signs of inflammation.

Notably, in its early stages, CD may not be visible on radiographs and may only manifest with disc swelling on MRI [16]. This is often associated with diffuse reactive edema on adjacent vertebral endplates. Additionally, contrast enhancement of the vertebral body is possible, due to the inflammatory response secondary to CD. This finding might be mistakenly attributed to an infectious lesion. To avoid this confusion, a CT scan should be performed to detect the calcification within the disc [1, 5].

Eventually, both pediatric and adult patients presenting with acute symptomatic calcific discitis will usually have a complete resolution of symptoms with conservative medical treatment, including analgesics and bed rest. Radiographic regression of the disc calcification, however, can remain incomplete, especially in adults [1].

## 4. Conclusion

Idiopathic calcific discitis is a rare cause of neck or back pain in adults. Our case shows that CD can even be associated with systemic signs of inflammation, including fever and increased CRP levels. The diagnosis of acute symptomatic CD must be recognized early enough to avoid disco-vertebral biopsy and subsequent antibiotics which are useless in this aseptic condition. Symptoms due to CD tend to resolve spontaneously with rest and analgesics after few days or weeks.
